# Promotion of Internet Users’ Aggressive Participation via the Mediators of Flow Experience and Identification

**DOI:** 10.3389/fpsyg.2022.836303

**Published:** 2022-05-02

**Authors:** Kuei-Feng Chang, Yu-Huang Huang, Wei-Chin Li, Shunjun Luo, Dong-Jenn Yang

**Affiliations:** ^1^School of Management, Guangzhou University, Guangzhou, China; ^2^Department of Marketing Management, Central Taiwan University of Science and Technology, Taichung, Taiwan; ^3^Department of Tourism, Food and Beverage Management, Chang Jung Christian University, Tainan, Taiwan; ^4^Department of Innovation and Strategy, School of Management, Guangzhou University, Guangzhou, China; ^5^College of Management, I-Shou University, Kaohsiung, Taiwan

**Keywords:** flow, identity, intrinsic motivation, habitual participation, social media, virtual communities

## Abstract

Social media users have increased rapidly in recent years; however, most are “silent users” who rarely share information online. To maintain social media companies’ stable operation and development, this research explored the effects of flow experience and identity formation on users’ intrinsic motivation to facilitate aggressive, spontaneous, and habitual participation in virtual communities. A total of 487 valid questionnaires were collected and underwent regression analysis. The results revealed that all three intrinsic motivations had a significant impact on social media participation, with social interaction exerting the strongest influence. Flow experience and identification had significant, partial mediating effects on the relationship between motivation and participation. Several suggestions were provided based on the results to help social media enterprises increase user participation. It is hoped that this research could facilitate in transforming users into habitual participants to keep the operations of the virtual community stable and enduring.

## Introduction

Social media users have grown explosively in recent years. Calvi and Jans (2010) defined “virtual communities” as online living circles that Internet users create on various platforms according to their interests and needs, through which they project their real life. A majority of Internet users belong to the “silent group” (Sun et al., 2014), who never share any information on social media (Preece et al., 2004). Social media companies may invest in converting the silent group into aggressive or habitual participants of the virtual community to maintain sustainable operations and developments.

The research on identification has two approaches: top-down (Bartels et al., 2007), which evaluates how organizations affect one’s organizational identification; and bottom-up (Harquail, 1998), which assesses personal thinking and feelings and personal identity negotiation toward the organization and its members. Virtual communities transcend space and race, where homogeneous members sharing similar interests and hobbies establish stable interpersonal relationships (Romm et al., 1997; Chang et al., 1999). Community identification is a bottom-up selective process involving identity negotiation, focusing on members’ individual experiences and feelings in the community and the stable relationships between them.

This research examines how users’ intrinsic motivation can be met via flow experience and identity formation to transform them into aggressive and habitual participants of virtual communities. Identification has two properties: noun (identity) described as a state of being and is considered stable; and verb (identify), which is a process subject to change and is dynamic (Cheney and Tompkins, 1987; Ashforth, 2001; Ashforth et al., 2008). Koufaris (2002) confirmed that mental flow impacts the browsing behavior and affective attitudes of Internet users. Games that consider mental flow in their design are likely to gain popularity and cause impulsive purchases (Hsu et al., 2012). Therefore, this research believes that the level of user involvement in a situation is related to primary motivation; identification is a static measuring variable and a dynamic process of shaping behaviors.

## Literature Review

### Need for Self-Presentation, Social Interaction, and Empowerment

The uses and gratifications approach analyzes the motivation for media use and the satisfaction of users’ needs. It also investigates the psychological and behavioral effects of mass communication (Katz et al., 1973). This holds that the force that drives social media use is users’ motivation to meet their needs or obtain a specific impact.

Most early mass media adopted a one-way persuasive communication; in contrast, social media allow users to create and exchange content with each other (Kaplan and Haenlein, 2010). Social media users improve their experience, collaboration, knowledge, and marketing (Constantinides, 2014). The early “new media” developed under Web 1.0 paid much attention to website browsing frequency or intensity (LaRose et al., 2001), while those under Web 2.0 feature content generation of users and interpersonal interaction (Daugherty et al., 2008). Due to continuous media use, web celebrities, such as bloggers and YouTubers, emerged and became opinion leaders of online worlds (Zhang et al., 2017), creating a more influential communication mode (Villanueva et al., 2008; Bao and Chang, 2014).

As diverse social media platforms expand, studies on the gratification of social media use based on the uses, and gratifications theory also increased. For instance, Whiting and Williams (2013) proposed ten uses and gratifications of social media. Some research added gratifications dedicated to specific social media like Facebook, Twitter, and Instagram (Sheldon and Bryant, 2016). However, this study believes that in Web 2.0, user gratification is closely related to social media’s instrumental characteristics, and its expected effects are an important dimension in generating one’s motivation to try it. Users’ interest in user-generated content (UGC), a social media characteristic, allows them to meet their need for self-presentation through content creation. Because of this need, users post various photos to express their personality and lifestyle (Sung et al., 2016) or share personal achievements with other users.

Furthermore, social media satisfies social interaction by providing a platform that integrates users’ information and promotes interpersonal communication. Through it, users can interact with others, improve social skills (Papacharissi and Rubin, 2000), and obtain support or recognition, fostering a sense of belonging (Buzeta et al., 2020). Lastly, social media meets the need for empowerment through activities, such as providing ways to respond and tag information, allowing them to have a positive understanding of their social activities within a virtual community. Social media users’ ability to express their opinions produces a sense of control and influence toward other groups (Muntinga et al., 2011; Tsai and Men, 2013).

### Aggressive Participation

Presently, lurkers account for 45.5–90% of social media users (Mason, 1999; Nonnecke, 2000; Nonnecke and Preece, 2000). The aggressive participation of users can be seen as an indicator for the stable development of virtual communities. Burnett (2000) divided participation into interactive and non-interactive. Non-interactive participation is a passive behavior; and is often displayed by “lurkers” who usually just browse websites or messages. Meanwhile, interactive participation is an active behavior exhibited by aggressive participants called “posters,” creating content, sharing knowledge, and responding to other community members (Ridings et al., 2006).

Wang and Fesenmaier (2004) classified community members’ participation into general and contributory behaviors. General behaviors satisfy the participant’s needs, such as browsing in virtual communities to look for information. In contrast, contributory behaviors benefit the whole community through knowledge and information sharing, helping each member develop the community. When lurkers read online content created by active users, they are regarded as indispensable “consumers” of the online community (Cranefield et al., 2015). Lurkers also have their own preferences and judgments, selecting content that interests them and seeking information with clear purposes (Gong et al., 2011). An active and successful community is closely related to the quality content created by its core members (Preece et al., 2004). Virtual communities’ value enhancement and sustainable development depend on aggressive participants (Rheingold, 1993). The long-term existence of virtual communities is related to the participation of community members (Algesheimer et al., 2005). Therefore, aggressive participants facilitate discussion, interaction, communication, knowledge transmission, and content creation.

Social media users actively partake in community activities when they want to contribute to new product design and when the activities are interesting (Fuller et al., 2006). Therefore, the most important motivators for participation are users’ curiosity and the need to exercise their capacity. Muniz and O’Guinn (2001) believed that users discuss the characteristics of the product, exchange ways to use it, and share user experience because they get a sense of achievement and pleasure from this process. These literature indicates that virtual community users participate in activities largely because of their interest, arousing their curiosity and inspiring them to utilize their knowledge and skills. Therefore, the need for self-presentation based on one’s interest is an important intrinsic motivation.

Members of virtual communities interact with others through knowledge sharing to acquire a sense of belonging and identity and create a positive interpersonal network (Hiltz and Wellman, 1997). The connection between users enables them to better understand each other, enhancing their social skills and establishing and maintaining relationships (Papacharissi and Rubin, 2000; Valenzuela et al., 2009; Lin and Lu, 2011). When community members establish a friendly relationship with others, they actively contribute information leading to interaction; thus, interpersonal relationships and communication are strengthened, and participation is promoted. Additionally, the need for social interaction by establishing relationships with others is an important intrinsic motivation for community members to participate in activities.

Chiu et al. (2006) found that community members share valuable information in virtual communities because they expect to win recognition and praise and enhance their influence in the community. Driven by the need for empowerment, social media users exert influence on groups (e.g., consumers, enterprises, and brands) by voicing their opinion and demanding to improve products, services and corporate policies (Muntinga, 2016), leading to more influential modes of communication (Villanueva et al., 2008; Bao and Chang, 2014). Community members improve their status and influence via discussion, interaction, knowledge sharing and content creation. Becoming an influencer allows users to meet their need for empowerment. Therefore, users’ influence and control over the community become the intrinsic motivation for aggressive participation. Based on the above discussions, the following are proposed:

H1a: Self-presentation exerts a positive impact on aggressive participation.

H1b: Social interaction exerts a positive impact on aggressive participation.

H1c: Empowerment has a positive impact on aggressive participation.

### Flow

Csikszentmihalyi (1975) first proposed the Flow Theory and defined flow as a strong emotional state in which people become engaged in an activity that nothing else seems to matter, ignoring irrelevant thoughts and merging their actions and awareness. Chen et al. (1999) believed that flow is a kind of psychological state; when individuals are fully engaged in an activity, they experience a change in perception of time, lose self-awareness, and enjoy great pleasure. This unconscious state produces automatic behavioral responses (Verplanken et al., 1997), which leads to habitually repetitive behavior that explains behavioral prediction, behavioral change and self-adjustment (Neal et al., 2006). Also, Kim (2004) noted that games developed with considerations to flow are more popular, and Novak et al. (2000) found that Internet users are more likely to have flow experiences when browsing, reading, and searching for needed information online. Hsu et al. (2012) investigated online shopping behaviors and found that flow encourages users to continue browsing and making purchases, even leading to impulsive buying. Chiang et al. (2011) indicated that users experience flow when playing online games, resulting in positive emotions.

Many scholars have different viewpoints on flow dimensions. Jackson and Marsh (1996) believed that flow consists of nine dispositions, while Hausman and Siekpe (2009) suggested only four dimensions. Novak et al. (2000) found that flow is linked to Internet users’ challenges, concentration, and skills. Kim et al. (2001) and Wang et al. (2012) summarized the research on flow and proposed three measurement constructs, namely: (1) concentration, (2) control, and (3) enjoyment. Concentration is the energy of one’s mind, which starts with allowing information to enter the brain (Csikszentmihalyi, 1990). Enjoyment refers to positive affective experiences, making individuals feel relaxed and experience a pleasure. Challenge refers to users’ perception of challenge-skill balance. A challenging task can fully engage people’s abilities, making it easier to concentrate on their task, which gratifies and gives them pleasure (Csikszentmihalyi, 1997).

Novak et al. (2003) found that flow experience occurs when individuals interact, encounter challenges, and utilize skills when browsing websites and pursuing new things. Moneta and Csikszentmihaiyi (1996) suggested that one’s perceived challenge-skill balance leads to flow experiences. Also, users perceive challenges in activities that differ depending on the situation. When individuals perceive that their ability equals the challenge of a task, the need for self-presentation urges them to take on the challenge, which leads to flow experiences.

Driven by the need for empowerment, users focus on the control of information channels to wield their influence. In a network-mediated environment, the interaction between users leads to a flow state, making them engage in the activity (Teng et al., 2012). When users strive to gain control and exercise influence over the community and lose their self-consciousness, they concentrate for a successful interactive process (Hartmann and Klimmt, 2006), allowing external information from the environment to enter their brain and give influential feedback via users’ behavior (Csikszentmihalyi, 1990).

Members of online brand communities perceive enjoyment when interacting with others, enabling them to strengthen their social connections (Schouten et al., 2007). Lu and Wang (2008) found that enjoyment perceived when playing online games makes users more reliant and confident in its utilization. Therefore, interaction among community members brings enjoyment, for which they reinforce their behavior. It is predicted that social media users get pleasure and enjoyment when interacting with others, which increases their participation in the community. Based on the above, the following are proposed:

H2a: Challenge mediates the need for self-presentation and aggressive participation.

H2b: Concentration mediates the need for empowerment and aggressive participation.

H2c: Enjoyment mediates the need for interaction and aggressive participation.

### Identification

In social identity theory, Tajfel (1978) defined “social identity” as part of an individual’s self-concept derived from his knowledge of his membership of a social group together with the value and emotional significance attached to that membership. Lee and Robbins (1998) defined it as social connectedness between a social group and its members. The social identity theory consists of three cognitive processes, namely (1) social categorization, (2) social identification, and (3) social comparison. Social categorization classifies individuals based on their understanding of the social world; social identification allows individuals to identify themselves as a group member; and social comparison is the process of comparing themselves with other groups in terms of prestige and social status (Tajfel, 1981). Overall, identification is described as perceived characteristics or roles (such as values, goals, and beliefs) of groups and a mixture of each member’s perceived characteristics (Postmes et al., 2006). Moreover, identification is a process in which individuals internalize the attributes of organizational identity, which is dynamic, subtle and vague (Ashforth et al., 2008).

The above statements revealed two basic elements for identification, namely cognition and evaluation. It also includes affective investment in consciousness and evaluation (Tajfel, 1982); thus, cognitive, affective, and evaluative dimensions all contribute to individuals’ social identification (Ellemers et al., 1999; Ashforth et al., 2008). Cognition refers to the consistency in the goals of individuals and group members. Affect means community members’ attachment to each other and a sense of belonging to the community. Evaluation refers to the evaluation of a community member. Marketing research has explored the behavior which brand community members display based on the social identity theory (Muniz and O’Guinn, 2001; McAlexander et al., 2002), approaching the dimensions that influence the behavior of small group brand community participants in terms of self-awareness, affective commitment and evaluative significance of membership (Bagozzi and Dholakia, 2006).

Rheingold (1993) noted that interaction resulting from shared interest among community members plays a crucial role in maintaining participation. Wang (2017) believed that virtual community platforms could improve its members’ sense of belonging and community identity through increased interaction, foster attachment between community members, and increase information sharing. Tianmiao (2018) believed that improving members’ identification greatly increases their participation, allowing them to win others’ acceptance and recognition via social interaction. In this process, they share values and create bonds between each other. As mentioned, social media users will spot what they have in common and establish close relationships with others via interaction because of their need for affiliation.

Individuals need the support and recognition of community members, especially when they mean to show their knowledge, wisdom, information, and skills. Hiltz and Wellman (1997) observed that community members like to share information with others, allowing them to interact to obtain a sense of belonging and identification. When social media users feel that they have a shared identity with community members, they tend to provide continuous positive affect for the community (Ashforth et al., 2008); their positive attitude is then reflected in their responses that highlight their knowledge, skills and abilities (Fishbein et al., 2001; Webb and Sheeran, 2006).

Huan (2019) noted that virtual communities need managers to protect the interests of community members, maintain community norms, and promote consensus among members; this creates a good atmosphere conducive to discussion and sharing. Based on the above, the following are proposed:

H3a: Cognition mediates social interaction and aggressive participation.

H3b: Affect mediates the need for self-presentation and aggressive participation.

H3c: Evaluation mediates the relationship between the need for empowerment and aggressive participation.

#### Social Influence Theory: Impact of Identification and Habit Formation on Aggressive Participation

Tajfel (1978) emphasized that people consider themselves as a group member based on perceived similarity with other members after social comparison or categorization. Identification is a process of self-reference; if the pre-existing motivation of social media users is highly consistent with the community, they will unconsciously integrate into it by participating in community activities and will have action-awareness merging with flow experience. Through this, they produce automatic responses, achieve goals, or reach a certain state (Verplanken et al., 1997), such as habitual participation or repetitive behavior. Further, individuals’ pre-existing motivation confirms a high degree of merging in the flow experience, strengthening individual thinking, feeling and evaluation via self-improvement, thereby creating content that community members expect from them. This way, a community identity comes into being, which leads to additional sense giving (Ashforth et al., 2008) and aggressive participation. However, if social media users fail to experience “loss of self-consciousness”, they still produce identification by conscious thinking and feeling (Ashforth, 2001). This is of great importance to turn ordinary users into aggressive participants through the processes of social influence.

The processes of social influence developed by Kelman (1961) explain how one’s emotion, opinion and behavior are influenced by others. It includes compliance (subjective norm), identification (social identity), and internalization (group norm) (Kelman, 1974). Compliance refers to one’s acceptance of others’ influence to win their recognition, receive encouragement, and avoid disapproval and punishment. Identification involves accepting others’ influence because they share similarities and play similar roles, to establish and maintain relationships. Internalization involves accepting others’ influence to keep their behaviors and beliefs consistent with their values and obtain intrinsic satisfaction (Kelman, 1963). Trenz et al. (2018) developed three social influence processes: positive word of mouth, peer use, and subjective norm, and explored their impact on user uncertainty and intent to use. Cheung and Lee (2010) investigated the intentional social action on online social networks, exploring the impact of subjective norm, group norm, and social identity on we-intention. These researches regard the three social influence processes as independent variables of the same level. However, this study believes that there should be a causal relationship between internalization, identification, and compliance under social influence.

As stated, social media under Web 2.0 require users to understand and adapt to its instrumental characteristics and learn how to operate it or get used to its influence; thus, compliance, a deliberate and unnatural behavior, is required to become new social media users. Without prior experiences, users decide to try new technology based on common subjective norms (Cheung and Vogel, 2013). Virtual communities often consist of highly homogeneous members who share interests and hobbies; they participate in activities based on their interests. In terms of similar motivations or values, individuals tend to think about what community member expects them to be via thinking, feeling and acting, which is a deliberate but natural beginning of the identification process. When using social media becomes inherent in users, they become attracted to community activities and even lose their self-consciousness. They may also experience gratification and pleasure when their values are consistent with the community, producing automatic and habitual behaviors leading to internalization. The former state is unconscious while the latter is conscious; only by changing the narratives of the existing motivation to adapt to situations can individuals get into the natural state that leads to flow experiences and repeated positive responses.

The above statements indicate two ways of cultivating aggressive participants. First, after social media users get the sensory signals of flow state, self-reinforcement catalyzes role identification that conforms to the community, leading to aggressive participation; second, motivation leads to flow experiences after modifying the narratives via community identification aggressive participation. Based on these, the following are proposed:

H4a: Social media users’ motivation leads to flow experiences, from which they identify with the community, leading to aggressive participation.

H4b: Social media users’ motivation triggers self-identification, for which they identify with the community, leading to aggressive participation.

## Materials and Methods

### Research Framework and Purpose

**FIGURE 1:**
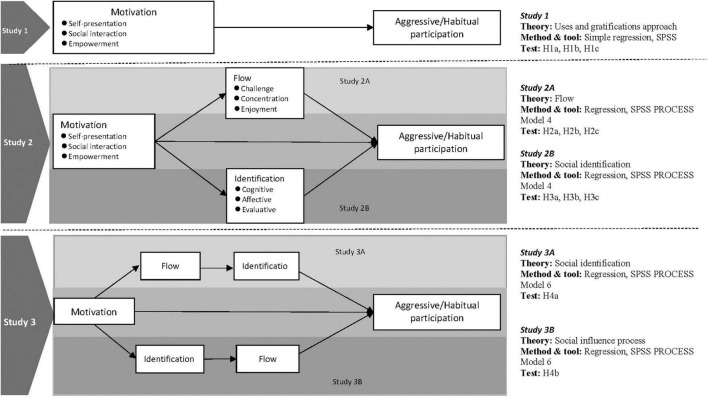
Research overview and conceptual framework.

As presented in the research framework (see Figure 1), this paper consists of three studies: Study 1 investigates the impact of motivation (need for self-presentation, social interaction and empowerment) on aggressive participation; Study 2 explores the impact of the motivation on responsive participation with flow (challenge, concentration, and enjoyment) and identification (cognitive, affective, and evaluative dimensions) as mediators; and Study 3 examined the influence path of aggressive participation from identification and habit formation processes.

### Questionnaire Design and Measurement

The questionnaire included two parts: (1) demographics of the respondents; and (2) the research variables. It consisted of 50 items scored using a seven-point Likert scale (*1* for *Strongly Disagree* and *7* for *Strongly Agree*). Regarding the number of questions in the questionnaire, the sample size should be 5–10 times the number of questions (Tinsley and Tinsley, 1987; Comrey, 1988). During the pilot study, 150 questionnaires were collected; the relevant validity and reliability analyses indicated that the questionnaire had good internal consistency (reliability) and validity (Please see Supplementary Appendix A for details). The final questionnaire was completed after revisions in its wordings. The 50 items included in the questionnaire are embedded into the manuscript as Supplementary Appendix A: Questionnaire Items and reliability and validity of Pre-test; at the same time, in Supplementary Appendix A, justification/support for items selection are also disclosed.

### Samples and Structure Analysis

In the questionnaire design management of this study, the general student communities are used as the pre-test, and the Weibo virtual communities are used as the main test objects; these two are not related to each other, and the pre-test samples are not included in the main test sample group. That is to say, convenient sampling was adopted in this study because there are different and diverse communities in the Weibo community. Therefore, more focused and in-depth research can be carried out with those specific communities. Users of the Chinese social media platform Weibo (a microblogging platform) were recruited as participants. Weibo has high user participation and a low threshold for community members to join. It has various communities such as brand, celebrity followers, and topic communities, in which many active members gather together. This makes community members on Weibo an effective and accurate source of data. The formal survey was launched 2 months after the completion of the pre-test. With the consent and assistance of bloggers, the questionnaire’s content was posted on different community platforms, and community members were free to choose whether to participate in the answering.

After 1 month of collection, 519 surveys were received, of which 32 were invalid (missing, contradictory, or repetitive responses), resulting in 487 valid questionnaires with a 93.8% effective recovery rate. The demographics are presented in Table 1.

**TABLE 1:** Participant demographics for pilot and formal surveys.

Variable		Pilot survey	Formal survey
			
		*N* = 150	*N* = 487
Gender	Male	48.8%	47.7%
	Female	51.2%	52.3%
Age	<20	12.5%	9.5%
	21–25	33.2%	55.9%
	26–30	24.8%	18.1%
	31–35	14.0%	9.2%
	>36	15.5%	7.3%
Education received	Junior high school and below	10.5%	5.8%
	Senior high school	16.5%	13%
	Junior college	22.5%	11.2%
	Colleges and universities	46.0%	64.7%
	Graduate school	4.5%	5.3%
			

## Analysis and Results

### Study 1: Impact of Motivations on Aggressive Participation

The SPSS 23.0 software was utilized to examine the influence of motivation for social media use (need for self-presentation, social interaction, and empowerment) on aggressive participation. The regression analysis results on Table 2 reveals that self-presentation (β = 0.214, *P* = 0.000 < 0.001), social interaction (β = 0.386, *P* = 0.000 < 0.001), and empowerment (β = 0.313, *P* = 0.000 < 0.001) had significant positive impacts on aggressive participation, supporting H1a, H1b, and H1c. Moreover, social interaction had the greatest influence on aggressive participation, followed by empowerment and self-presentation.

**TABLE 2:** Path coefficients and verification results for Study 1.

Hypothesis	Independent variable	Dependent variable	Beta	*P*	Results
H1a	Self-presentation		0.214	***	Supported
H1b	Social interaction	Aggressive participation	0.386	***	Supported
H1c	Empowerment		0.313	***	Supported

***P < 0.001.

### Study 2: Mediating Effect of Flow and Identification

Model 4 in the PROCESS software package (Hayes, 2013) was adopted for regression analysis to examine the mediating effects of flow (Study 2A) and identification (Study 2B).

As shown in Table 3, the mediating effect of challenge on the relationship between self-presentation and aggressive participation was significant (95% CI of 0.1144–0.2076). The value of the mediating effect was 0.1551, accounting for 56.22% of the total effect (i.e., 0.2759), indicating partial mediation. Moreover, the mediating effect of enjoyment on the relationship between social interaction and aggressive participation was significant (95% CI of 0.0768–0.1669). The value of the mediating effect was 0.1403, accounting for 37.01% of the total effect (i.e., 0.3790), indicating partial mediation. Lastly, the mediating effect of concentration on the relationship between empowerment and aggressive participation was significant (95% CI of 0.1005–0.1864). The value of the mediating effect was 0.1403, accounting for 37.01% of the total effect (i.e., 0.3790), indicating partial mediation. Thus, H2a, H2b, and H2c are all supported.

**TABLE 3:** The mediating effect of flow.

H	Path	Effect	Effect size	Boot SE	Bootstrap 95% CI		Percentage	*P*	Mediation
					
					Lower limit	Upper limit			
H2a	Self-presentation → challenge → aggressive participation	Total	0.2759	0.041	0.1958	0.356	100.00%	***	Partial
		Direct	0.1208	0.0403	0.0028	0.1999	43.78%	**	
		Indirect	0.1551	0.0232	0.1144	0.2076	56.22%	***	
H2b	Social interaction → enjoyment → participation	Total	0.4917	0.0379	0.4172	0.5661	100.00%	***	Partial
		Direct	0.3747	0.0391	0.298	0.4514	76.20%	***	
		Indirect	0.117	0.0228	0.0768	0.1669	23.80%	***	
H2c	Empowerment → concentration → participation	Total	0.379	0.0373	0.3058	0.1522	100.00%	***	Partial
		Direct	0.2387	0.0385	0.1631	0.3143	62.98%	***	
		Indirect	0.1403	0.022	0.1005	0.1864	37.01%	***	

*P < 0.05, **P < 0.01, and ***P < 0.001.

As shown in Table 4, the mediating effect of the affective dimension on the relationship between self-presentation and participation was significant (95% CI of 0.1958–0.3560). The value for its mediating effect was 0.1401, accounting for 50.77% of the total effect (0.2759), suggesting partial mediation. Further, the mediating effect of cognitive dimension on the relationship between social interaction and participation was significant (95% CI of 0.1515–0.2397). The value for the mediating effect was 0.1948, accounting for 39.62% of the total effect (i.e., 0.4917), indicating partial mediation. Lastly, the mediating effect of the evaluative dimension on the relationship between empowerment and participation was significant (95% CI of 0.1121–0.1998). The value of the mediating effect was 0.1542, accounting for 49.69% of the total effect (i.e., 0.3790), indicating partial mediation. Thus, H3a, H3b, and H3c are all validated.

**TABLE 4:** The mediating effect of identification.

H	Path	Effect	Effect size	Boot SE	Bootstrap 95% CI		%	*P*	Mediation
					
					Lower limit	Upper limit			
H3a	Self-presentation → Affective dimension → Participation	Total	0.2759	0.0408	0.1958	0.356	100.00%	***	Partial
		Direct	0.1358	0.0388	0.0996	0.1893	49.22%	***	
		Indirect	0.1401	0.0232	0.0996	0.1893	50.77%	***	
H3b	Social interaction → Cognitive dimension → Participation	Total	0.4917	0.0379	0.4172	0.3717	100.00%	***	Partial
		Direct	0.2969	0.0381	0.2221	0.3717	60.38%	***	
		Indirect	0.1948	0.022	0.1515	0.2397	39.62%	***	
H3c	Empowerment → Evaluative dimension → Participation	Total	0.379	0.0373	0.3058	0.4522	100.00%	***	Partial
		Direct	0.2248	0.0.52	0.1558	0.2938	59.31%	***	
		Indirect	0.1542	0.0224	0.1121	0.1998	49.69%	***	

*P < 0.05, **P < 0.01, and ***P < 0.001.

### Study 3: Impact of Identification and Habit Formation Processes on Aggressive Participation

To verify the identification process (Study 3A) and habit formation process (Study 3B) of aggressive participation, Model 6 in the PROCESS software package (Hayes, 2013) was adopted.

**FIGURE 2:**
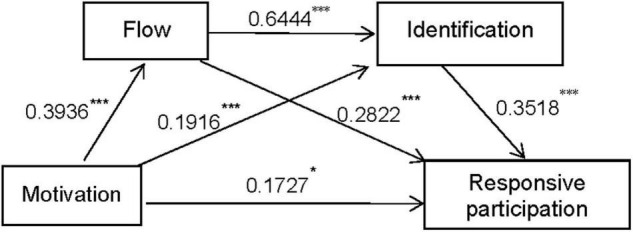
Mediation path analysis of flow and identification. *P < 0.05, **P < 0.01, and ***P < 0.001.

In Study 3A, the motivations were set as independent variables (X), aggressive participation as dependent variable (Y), flow as first mediator (M1) and identification as second mediator (M2); the path was Motivation → Flow → Identification → Aggressive participation. The empirical results showed that the motivations had a significant impact on aggressive participation. The indirect effect of flow (*b* = 0.1183, BootSE = 0.0298, BootCI95 = [0.0642, 0.1766]) and the indirect effect of identification (*b* = 0.0842, BootSE = 0.0206, BootCI95 = [0.0475, 0.1271]) were significant. The serial mediation model also revealed that the indirect effect (*b* = 0.1158, BootSE = 0.0168, BootCI95 = [0.0859, 0.1509]) was significant (Please see following Figure 2 for illustration).

**FIGURE 3:**
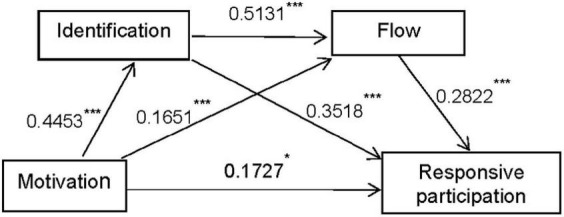
Mediation path analysis of identification and flow. *P < 0.05, **P < 0.01, and ***P < 0.001.

In Study 3B, the motivations were set as independent variables (X), aggressive participation as dependent variable (Y), identification the first mediator (M1) and flow the second mediator (M2); the path was Motivation → Identification → Flow → Aggressive participation. The empirical results showed that motivation had a significant impact on aggressive participation. The indirect effect of identification (*b* = 0.2001, BootSE = 0.0288, BootCI95 = [0.1451, 0.2581]) and the indirect effect of flow (*b* = 0.0448, BootSE = 0.0116, BootCI95 = [0.0366, 0.1168]) were significant. The serial mediation model revealed that the indirect effect (*b* = 0.0448, BootSE = 0.0116, BootCI95 = [0.0240, 0.0697]) was also significant (Please see following Figure 3 for illustration).

The results of the two serial mediation models revealed that H4a and H4b are validated. The path coefficients were then multiplied and the results showed that the product of the first path (0.0892) was larger than that of the second path (0.0645); thus, in terms of triggering positive responses, the identification process path has a stronger effect on aggressive participation than the habit formation process path (Please see following Table 5 for details).

**TABLE 5:** Verification of chain mediating effects.

Hypothesis	Path	Effect size	Bootstrap 95% CI	Verification
H4a	Motivation → Flow → Identification → Responsive participation	0.1158	[0.0859, 0.1509]	Supported
H4b	Motivation → Identification → Flow → Responsive participation	0.0448	[0.0240, 0.0697]	Supported

## Conclusion and Suggestion

### Conclusion

Self-presentation, social interaction, and empowerment all have a significant impact on responsive participation. When social media users are motivated to share their abilities, establish relationships, and exercise influence, they are more encouraged to participate in social media activities. Among the three motivations, social interaction has the strongest effect on participation, indicating that social media users have a strong desire to establish good relationships with community members. The impact of empowerment on responsive participation is the second strongest, indicating that posters are content creators and influencers in an online community.

For many social media users, social interaction is their primary motivation. If social media users only click, browse, and read, they merely act as information receivers. When they find pleasure in interacting with others or realize that they share similar goals, they will recognize the necessity of bidirectional or multidirectional participation. Moreover, content creation is conducive to self-presentation. If associated challenges provide them with pleasure and a sense of achievement, they will invest more time and energy into the community. Similarly, social media users are empowered to post events that they consider meaningful; thus, empowerment produces a positive learning effect, obtaining support from the community through an increase in followers, likers, and reposts.

For lurkers, aggressive participation promotes their identity, which can be seen as a habit formation process. The difference between identification and habit formation processes lies in merging one’s awareness and actions. The comparison of path coefficients indicates that when users are immersed in social media use (unconscious), they will identify with the community leading to a higher path coefficient of aggressive participation than that of community identification (conscious), after which the flow state can be reached. The corresponding results are consistent with Ashforth et al. (2008), suggesting that identification involves affective dimensions; however, its specific form depends on the situation, and it often changes along with the organization. When a community cultivates the habit of its users, it is hard to know the extent to which their awareness and action merge due to individual differences (Brewer and Gardner, 1996). Although individuals have already produced affective identification, they can only internalize their participatory behaviors into a habit with the situations and their gratifications. This is consistent with the habit formation by Kelman (1961), which explains why some communities reward players with virtual tokens to encourage them to take challenges regularly. Through concentration, players can complete the objectives that give them enjoyment, leading to habitual behavior patterns.

### Suggestions

Social media users’ need for social interaction refers to their intention to establish cooperative relationships. Therefore, social media enterprises must design various interactive channels, such as chatrooms, live streaming channels, and comment sections, to strengthen and establish close communication and interaction between users.

The need of empowerment indicates that users want to exert influence because they value social status and prestige. Therefore, social media enterprises are advised to set up scoring systems, reward points, and user promotion and rankings to satisfy their desire for prestige. Moreover, they can be invited to serve as forum moderators and community administrators by giving them more authority to operate.

Self-presentation entails accomplishing challenging tasks to obtain a sense of achievement. Enterprises are advised to respond to their participation positively, such as appraising, highlighting, and recognizing their content, to bolster their morale.

Social media enterprises need to provide users with positive situational experiences to trigger a flow state. For instance, they can design challenging activities, push for quality information or up-to-date news, and interact with their users via games to give them enjoyment and encourage them to continue using the app. As for identification, enterprises can strengthen their relationship with users by hosting activities dedicated to core members, facilitating a sense of community belongingness. As for aggressive participants, social media enterprises can give virtual tokens representing their identity in their profiles. Moreover, they can increase the number of followers as a threshold, or establish a payment mode (such as paid live streaming), so that community resources can be directed to the posters. This way, the relationship between the community and users can be transformed into a sustainable business partnership.

### Theoretical Contribution and Practical Implications

This study investigated primary motivation and its effect on transforming lurkers into posters who have their behavior patterns. Empowerment, though less explored, is a source of motivation for social media use and could lead to the emergence of online opinion leaders and business models. Consequently, this study assumed that the cultivation of lurkers into posters is a dynamic process. According to Ashforth et al. (2008, p. 340), “a process model of identification should account for this dynamism, explicating the intense episodes that require conscious, deliberate decisions that serve to either solidify or transform identities.” This present research set flow as an antecedent that influences one’s identification in social media. Moreover, the correlation between the subconstructs of flow and identification and primary motivations were explored, which explains the model of aggressive participation; it supplemented the bottom-up type approach of identity theories and responded to the suggestions of Ashforth et al. (2008).

Lastly, to utilize social media’s UGC to increase the number of posters, community moderators should expect members to participate aggressively in activities and turn them into habitual participants. This research explained the causal relationship between the processes of influence and habit formation based on the difference of the three processes of the social influence theory.

In terms of managerial implications, for users who need self-presentation, the company implements a positive response to the participation behavior of such users on the one hand, highlights these displayed contents, and make appropriate recommendations to more users so that users can feel their creations being recognized and welcomed of their creations in social media.

For users who interact with each other, in addition to designing interactive communication channels and media in social media, enterprises can also strengthen the frequency of communication and interaction between users, thereby forming a close connection between users, and thus encouraging users to communicate with each other. For example, users can collaborate to create high-quality content, such as videos, to enhance their friendship with each other during the process of creation.

For users who prefer empowerment and authorization, enterprises can design points acquiring system, virtual rewards, level promotion, user rankings and other functions to achieve users’ satisfaction and relevant experiences with their participation and prestige. Thus, users can realize that they are interacting with users by granting more operation permissions to meet their needs for empowerment and authorization.

### Limitation and Future Scope

In terms of research limitations, the sample size of this study is slightly insufficient. According to the research needs, this research design includes 10 variables and 50 items; based on the number of questions in the questionnaire; the sample size should better be 5 to 10 times the number of questions (Tinsley and Tinsley, 1987; Comrey, 1988). However, Sudman (1976) also pointed out that if the research object is a regional study, selecting 500–1,000 people is recommended. Although the pre-test sample (*n* = 150) results in this study showed that the questionnaire itself had good reliability and validity, the valid sample of the main test (*n* = 487) failed to exceed 500 after deleting the invalid sample. Therefore, it is suggested that future studies can collect a higher amount of questionnaires.

In addition, this study takes various social media software to research to illustrate the influence of the motivation of community members on their active participation in social media. Therefore, it is suggested that future research can focus more on a specific social media, and have certain improvement targets such as understanding (1) community members’ preferences for social media marketing incentives, (2) differences in the motivational or hierarchical revolution of the relational network of the users, and (3) personal self-concepts’ impacts on positive behavior and psychology that may lead to more objective and effective recommendations for specific social media managers.

## Data Availability Statement

The original contributions presented in the study are included in the article/Supplementary Material, further inquiries can be directed to the corresponding author/s.

## Author Contributions

K-FC: making sure the conceptual research framework of this study, confirmation and inferring of hypothesis, abstracts, and introduction, and supervising on the progress of the research. Y-HH and W-CL: revising the manuscript according to the editor and reviewers’ comments, final proofs’ editing, writing of managerial implications, research limits, and future studies. SL: translation, checking, formation, submission, and literature review. D-JY: data collection and analysis, statistical analysis, conclusion presentation, and mainly focusing on the method parts. All authors contributed to the article and approved the submitted version.

## Conflict of Interest

The authors declare that the research was conducted in the absence of any commercial or financial relationships that could be construed as a potential conflict of interest.

## Publisher’s Note

All claims expressed in this article are solely those of the authors and do not necessarily represent those of their affiliated organizations, or those of the publisher, the editors and the reviewers. Any product that may be evaluated in this article, or claim that may be made by its manufacturer, is not guaranteed or endorsed by the publisher.
