# Supplementary material for: Promotion of Internet Users’ Aggressive Participation via the Mediators of Flow Experience and Identification
Source: Front Psychol. 2022 May 2;13:836303. doi: 10.3389/fpsyg.2022.836303 (PMC9108352; doi:10.3389/fpsyg.2022.836303)
Supplement: Supplementary file 1 [file Data_Sheet_1.pdf]

Appendix A Questionnaire Items and reliability and validity of Pre-test

Measurements	Cite and design reference sources	Factor loading	Cronbach’s α
Justification/support for items selection on dimension (Self-Presentation): Sung et al. (2016)			
SP1	I can show my hobbies through social media.	.875	.966
SP2	I can express my ideas through social media.	.891	
SP3	I can state my opinions through social media.	.872	
SP4	I can present my tastes through social media.	.854	
SP5	I can provide my reviews through social media.	.889	
Justification/support for items selection on dimension (Social Interaction): Sung et al.(2016)			
SI1	I want to know what people think by using social media.	.830	.972
SI2	I want to communicate with others by using social media.	.778	
SI3	I want to share information by using social media.	.813	
SI4	I want to make more friends by using social media.	850	
SI5	I want to build friendly relationships by using social media.	.889	
Justification/support for items selection on dimension (Empowerment): Tsai & Men (2013)			
EM1	I hope to become a social influencer on social media.	.845	.956
EM2	I hope to become a rule-maker on social media.	.866	
EM3	I hope to become an authoritative speaker on social media.	.850	
EM4	I hope to become a topic leader on social media.	.839	
EM5	I hope to become a professional representative on social media.	.865	
Justification/support for items selection on dimension (Concentration of Flow): Moon & Kim (2001)			
CF1	I am focused in the process of using social media.	.809	.962
CF2	I am concentrated on the process of using social media.	.821	
CF3	I am absorbed in the process of using social media.	.870	
CF4	I am preoccupied with the process of using social media.	.799	
CF5	I am wholehearted in the process of using social media.	.855	

Justification/support for items selection on dimension (Enjoyment of Flow): Cho et al. (2011)			
EN1	Social media makes me happy.	.870	.981
EN2	Social media makes me pleased.	.868	
EN3	Social media makes me amused.	.849	
EN4	Social media makes me enjoy it.	.851	
EN5	Social media makes me interested.	.860	
Justification/support for items selection on dimension (Challenge of Flow): Jackson & Eklund (2004)			
CH1	I can master a rich set of functions of social media.	.785	.959
CH2	I can skillfully operate social media.	.822	
CH3	I can face the challenges of social media change.	.908	
CH4	I can keep pace with the new activities on social media.	.872	
CH5	I can overcome the difficulties in using social media.	.899	
Cognition of Identification Bagozzi et al. (2000)；Ellemers（1993）			
CI1	I have a common direction with the community.	.824	.975
CI2	I have a similar ideal with the community.	.836	
CI3	I have a common expectation with the community.	.807	
CI4	I have a similar goal with the community.	.826	
CI5	I have a shared vision with the community.	.826	
Justification/support for items selection on dimension (Affect of Identification): Bagozzi et al. (2000); Ellemers(1993)			
AI1	I am fond of the community.	.793	.968
AI2	I feel dependent on the community.	.800	
AI3	I feel friendly toward the community.	.757	
AI4	I feel genial toward the community.	.811	
AI5	I feel delighted toward the community.	.785	
Justification/support for items selection on dimension (Evaluation of Identification): Bagozzi et al. (2000); Ellemers (1993)			
EI01	I think the community I am currently in is worth supporting.	.763	.961

EI02	I think the community I am currently in is satisfying.	.805	
EI03	I think the community I am currently in is worth praising.	.849	
EI04	I think the community I am currently in is worth recognizing.	.847	
EI05	I think the community I am currently in is worth approving.	.805	
Justification/support for items selection on dimension (Aggressive Participation): Koh & Kim (2004)			
AP1	I will share what I know on social media.	.852	
AP2	I will discuss this with other users on social media.	.852	
AP3	I will respond to other users' topics on social media.	.897	. .974
AP4	I will participate in the activities announced by the community on social media	.897	
AP5	I will forward information from others on social media.	.879	
